# Side Effects of COVID-19 Vaccines in Pregnant and Lactating Mexican Women and Breastfed Infants: A Survey-Based Study

**DOI:** 10.3390/vaccines11081280

**Published:** 2023-07-25

**Authors:** María Elena Camacho Moll, Ana María Salinas Martínez, Benjamín Tovar Cisneros, Juan Ignacio García Onofre, Gloria Navarrete Floriano, Mario Bermúdez de León

**Affiliations:** 1 Department of Molecular Biology, Northeast Biomedical Research Center, Mexican Institute of Social Security, Monterrey 64720, N.L., Mexico; mario.bermudez@imss.gob.mx; 2 Center for Molecular Diagnosis and Personalized Medicine, Health Sciences Division, Universidad de Monterrey, San Pedro Garza García 66238, N.L., Mexico; 3 Epidemiologic and Health Services Research Unit, Mexican Institute of Social Security, Monterrey 64360, N.L., Mexico; 4 School of Public Health and Nutrition, Autonomous University of Nuevo Leon, Monterrey 64460, N.L., Mexico; 5 School of Biological Sciences, Autonomous University of Nuevo Leon, Monterrey 66455, N.L., Mexico; btovar@exatec.tec.mx; 6 Family Medicine Unit No. 64, Mexican Institute of Social Security, Santa Catarina 66358, N.L., Mexico; netherlands06@hotmail.com (J.I.G.O.); gloria.navarretef@imss.gob.mx (G.N.F.)

**Keywords:** SARS-CoV-2 vaccine, COVID-19 vaccine, side effects, pregnancy, breastfeeding

## Abstract

COVID-19 vaccines’ safety has been extensively studied; however, further analysis is required in pregnant women, nursing mothers, and breastfed infants. Our aim was to compare the extension and severity of self-reported COVID-19 vaccine side effects in pregnant and breastfeeding women, and breastfed infants. In this cross-sectional study, COVID-19-vaccinated subjects were enrolled using an online survey in Mexico. Women were classified by pregnancy and breastfeeding status at the time of vaccination (*n* = 3167). After the first or only dose, there was a trend toward fewer systemic effects in pregnant women (*p* = 0.06). BNT162b2 (Pfizer–BioNTech) had a higher frequency of local symptoms in pregnancy. Lactating women experienced fewer local symptoms after the first or single dose (*p* = 0.04) and the opposite occurred after the second dose (*p* = 0.001). ChAdOx1 (AstraZeneca) increased the chances of developing both local and systemic symptoms after the first dose but decreased them after the second dose. The severity was similar across groups, although the result of lack of association in pregnancy requires studies with a larger sample size. Irritability was the most reported symptom in breastfed infants. This study contributes to the knowledge about the side effects in pregnant and lactating women, and breastfed babies.

## 1. Introduction

COVID-19 is a respiratory disease that paralyzed the world for several months by causing the death of more than six million people. The speed at which we travel the world in the 21st century caused a pandemic in just three months [1]. Furthermore, today’s technology allows us to trace the virus from its first reports in a seafood market in Wuhan, China to the first case in every country. We now know the nucleotide sequence of the virus [2] and therefore vaccines were developed at unprecedented rates. In March 2021, a year after the first COVID-19 case in December 2019, vaccination started. In Mexico, from December to February 2021, vaccination was prioritized for healthcare workers, followed by the population aged 60 and over. From April to May 2021, schoolteachers and people aged 50 to 59 were vaccinated. From May to June 2021, people aged 40 to 49 were vaccinated, and finally from June 2021 to present, the remaining population was vaccinated [3]. To date, 13,337,416,815 doses have been administered worldwide, of which 222,580,922 doses have been administered in Mexico [1]. Pregnant women have an elevated risk of severe COVID-19 [4] and traces of mRNA vaccines have been detected in breast milk [5,6]; therefore, studying the safety of COVID-19 vaccines in these groups of the population is imperative, given they are already being exposed. Plenty of studies have reported the safety and efficacy of COVID-19 vaccines [7,8,9,10,11,12,13], but these are all clinical trials conducted on healthy participants with no participation of pregnant or breastfeeding women. However, observational studies had shown vaccines were safe in these populations, several of which were based on self-reported symptoms post-vaccination [8,11,12,13,14,15,16,17,18,19,20,21,22,23,24,25,26,27,28,29,30,31,32,33,34,35,36,37,38,39,40,41,42]. We have previously reported the side effects caused by seven COVID-19 vaccines in the Mexican population [3]. However, the extension and severity of side effects was not compared between pregnant women and non-pregnant/non-lactating women. Furthermore, in the present study we describe the extension and severity of side effects reported by breastfeeding women compared to non-pregnant/non-lactating women, which has not been shown before. Pregnant women’s vaccination began in May 2021 in Mexico, and antibodies have been reported in the newborn [14] because they are transferred through the breast milk [19,22,43]. Additionally, local and systemic symptoms are expected in pregnant and lactating women [16], and breastfed infants [21]. Reports on the extension and severity of COVID-19 vaccine side effects in pregnant and lactating women are few. A recent literature review showed five studies describing post-vaccination side effects in lactating women [5,21,43,44,45,46] and three in breastfed infants [5,21,46]. However, none of them corresponds to the Mexican population. Shimabukuro and colleagues (2021) have reported preliminary findings on mRNA COVID-19 vaccine safety in pregnant women [18], and several agencies only recommend the use of BNT162b2 (Pfizer–BioNTech) and mRNA-1273 (Moderna) [47]. There are reports from several countries indicating pregnant women’s attitudes toward COVID-19 vaccination during pregnancy, which demonstrate that fear of the side effects, lack of safety data, and mistrust in vaccines are the most reported reasons against vaccination [48,49,50,51,52]. Pregnant women not being included in the clinical trial might have built confusion, which led to mistrust in the vaccine even when updated evidence was being published. Social medial and political concerns have also been reported to lead to a negative perception towards vaccination.

In the present study we compared the extension and severity of self-reported COVID-19 vaccine side effects in pregnant and breastfeeding women. We also explored the presentation of side effects in breastfed infants.

## 2. Materials and Methods

This is a secondary analysis performed after a cross-sectional study conducted in Mexico from 12 August to 3 September 2021 (5 months after starting vaccination in the population other than healthcare workers, and 3 months after starting vaccination in pregnant women) focused on the female population. The database consisted of participants with a vaccine against COVID-19, mainly from Nuevo Leon, Mexico City, and the State of Mexico. The snowballing sampling technique was used for recruiting subjects. The survey was shared on social media platforms such as Facebook and WhatsApp; therefore, it was not possible to estimate the number of invited people. Word-of-mouth campaigns were also used with no proportional quotas by sociodemographic variables or by type of vaccine. Participants did not receive any kind of incentive. The self-applied electronic survey was designed in Spanish with the software tools QuestionPro (Survey Analytics LLC, San Francisco, CA, USA) and Google Forms (Google, Mountain View, CA, USA). The survey took an average of 5 min to complete [3]. For the present study, a minimum sample size of 385 was calculated based on a 50% estimated frequency of at least one local or systemic side effect assuming a 95% confidence level with a precision of 5% [53,54]. A total of 3204 women were eligible: 110 pregnant, 363 breastfeeding, and 2694 non-pregnant/non-breastfeeding; a total of 37 had to be excluded for simultaneously presenting pregnancy and lactation. No sample size was estimated for breastfed infants and the analysis of side effects in this population was exploratory. The protocol was approved by the Institutional Review Board (or Ethics Committee) of the Mexican Social Security Institute (R2021-1909-106). The study followed the Declaration of Helsinki’s guidelines for research on human subjects, and all the participants signed their informed consent digitally before filling in the questionnaire. Participation was entirely voluntary, and withdrawal was allowed at any time without the need to justify the decision. There was no personal data collected that might enable the retrospective identification of the participant. 

### 2.1. Study Variables

The variables included in the present study were vaccine data, type and number of doses, use of medication prior to vaccination to prevent symptoms (yes, no), and type of side effect (the participant chose from a list made with short-term side effects reported in the literature). The side effects were self-reported, similar to many observational studies [8,20,21,22,23,24,25,26,27,28,29,30,31,32,33,34,35,36,37,38,39]. Dose 1 and 2 side effects were questioned in separate sections. The extension of the side effect was categorized as none, local (e.g., pain at the injection site), systemic (e.g., fever), or both. The severity of the side effect was classified as absent, mild, moderate, or severe, according to the need for taking medication to relieve symptoms, interruption of activities or missing work, having to visit a doctor, an emergency room, or required hospitalization [3] (Table S1). Information on severity was available only for the first or only dose. 

Other variables were comorbidity (prediabetes, diabetes, hypertension, chronic renal failure, chronic obstructive lung disease, asthma, an immune disease, cancer, a cerebrovascular disease, other), history of allergies, COVID-19 infection (had symptoms consistent with COVID-19 disease and was positive with PCR or rapid nasal swab antigen test), sex, age, schooling, occupation, place of residence, and smoking. The nutritional status was assessed using a validated body mass index–body size pictorial method [55] that indicated low weight (shape A), normal weight (shapes B and C), overweight (shape D), and obesity (shapes E to J). 

### 2.2. Statistical Analysis

Frequencies were obtained for the categorical variables, and means and standard deviations were obtained for the non-categorical variables. The chi-square test was used to compare the frequency distribution of sociodemographic, medical, and vaccination history characteristics among non-pregnant/non-lactating, pregnant, and lactating women. The Fisher’s exact test or the chi-square test was used to compare the frequency distribution of the extension of vaccine side effects between non-pregnant/non-lactating and pregnant or lactating women. A multivariate ordinal logistic regression was used for estimating odds ratios (OR) and 95% confidence intervals (CI) between the type of vaccine (independent variable) and the extension of symptoms (dependent variable; 0 = absence of side effects, 3 = presence of local and systemic side effects) adjusting for potential confounders in each population of interest (pregnant and lactating), separately. Additionally, for severity of symptoms, 0 = absent and 3 = severe.

## 3. Results

The population mean age was 38.6 ± 10.7 years old. More than half of the participants had undergraduate and graduate degrees, 69.6% were employed or self-employed, 12% smoked, 50.7% were obese, 19.5% had at least one comorbidity, 6.5% had hypertension, 4.4% had pre/diabetes, and 32.8% had a history of allergies. The most frequent vaccinations were BNT162b2 (Pfizer–BioNTech) (39%) and ChAdOx1 (AstraZeneca) (29.7%), followed by Ad5-nCoV (CanSinoBIO) (15.1%), CoronaVac (Sinovac Life Sciences) (7.3%), Gam-COVID-Vac (Gamaleya’s Sputnik V) (5.2%), Ad26.CoV2.S (Johnson & Johnson/Janssen) (2.4%), and mRNA-1273 (Moderna) (1.3%). A previous COVID-19 infection was present in 29.1%. We also collected data from breastfed infants (see Supplementary Tables S4 and S5).

### 3.1. Pregnant vs. Breastfeeding vs. Non-Pregnant/Non-Breastfeeding Women’s Side Effects

Pregnant and breastfeeding women differed from non-pregnant/non-breastfeeding women in sociodemographic, medical, and vaccination history characteristics. There were more pregnant and lactating women aged 30 to 39 years. There were fewer smokers among the pregnant and breastfeeding than non-pregnant/non-breastfeeding women. They also had fewer comorbidities and used less preventive medication than non-pregnant/non-breastfeeding women. BNT162b2 and ChAdOx1 were the most common vaccines in pregnant and breastfeeding women (Table 1). 

### 3.2. Extension of Side Effects in Pregnant Women

There was a trend toward fewer systemic effects after the first or only dose in pregnant compared to non-pregnant/non-breastfeeding women. They experienced fewer headaches, fevers, and hot flashes, and less muscle pain, fatigue or tiredness, malaise, dizziness, and chest pain. After the second dose, there was no difference in symptom presentation (Table S2). BNT162b2 had a higher frequency of local symptoms after the first and second dose. ChAdOx1 and Gam-COVID-Vac increased the chances of experiencing both local and systemic symptoms after the first dose compared to BNT162b2 (Table 2). 

### 3.3. Extension of Side Effects in Breastfeeding Women

There were fewer local symptoms and more headaches, fatigue, bone or joint pain, nausea, and dizziness in lactating women after the first or only dose. In contrast, local symptomatology predominated after the second dose, especially arm and injection-site pain (Table S3). BNT162b2 had more local effects and ChAdOx1 increased the chances of experiencing both local and systemic effects after the first and second dose compared to BNT162b2 (Table 3). 

### 3.4. Severity of Side Effects in Pregnant and Breastfeeding Women

The severity after the first or only dose was similar between pregnant, lactating, and non-pregnant/non-breastfeeding women (Table 4). After the first dose, ChAdOx1 and Gam-COVID-Vac slightly increased the chances of moderate/severe symptoms in pregnant and lactating women, (Table 5). 

### 3.5. Exploratory Results on the Presentation of Side Effects in Breastfed Infants 

More symptoms were reported in infants aged 4.1–6 months (28%) and in those who received the CoronaVac vaccine (23%) (Table S4). Secondary symptoms were present in 11% of breastfed infants; irritability was the most mentioned effect (Table S5).

## 4. Discussion 

Pregnant women are at a higher risk of severe COVID-19 [56,57,58]. Therefore, they must obtain a vaccination. One reason why pregnant women are hesitant to receive a COVID-19 vaccination is the lack of safety data from the clinical trials, where pregnant women were not included. Social media has been pointed out as a source of misinformation [50,59], and politics has also been involved in shaping women’s perceptions about COVID-19 vaccination [60]. The present study provides evidence for the short-term safety of the COVID-19 vaccine in pregnant and lactating women.

### 4.1. Extension of Side Effects during Pregnancy

ChAdOx1 and Gam-COVID-Vac increased the chances of both local and systemic symptoms after the first dose. We listed a total of five types of local and 39 types of systemic symptoms in pregnancy. The most common symptoms were pain in the arm, injection-site pain, headaches, muscle pain, the desire to sleep, fatigue or tiredness, and a lack of energy. This was consistent with what has been reported in the literature [16,17,61,62]. The most cited study on self-reported side effects in pregnancy was published in 2021 [18], where injection-site pain, fatigue, headaches, and myalgia were the most commonly reported symptoms in 35,691 pregnant women who received mRNA-1273 and BNT162b2. Side effects were more frequently reported after the second dose. 

### 4.2. Extension of Side Effects during Breastfeeding

Lactating women experienced fewer local symptoms after the first or only dose. However, the opposite occurred after the second dose, and arm and injection-site pain were common. Another study found that the frequency of reactions was higher after the second dose [46]. Pain, redness, and swelling at the injection site have been frequently reported after the second dose [45]. We found that ChAdOx1 registered higher chances for both local and systemic side effects after the first dose, but these chances were reduced after the second dose compared to BNT162b2. Golan and colleagues found a higher presentation of any injection-site symptoms with mRNA-1273 than BNT162b2, after the first and second dose [5]. 

### 4.3. Severity of Side Effects during Pregnancy and Breastfeeding

We found that breastfeeding status had no effect on severity. The statistically non-significant result could be due to the small sample size of pregnant women. Therefore, it should be considered as inconclusive. Studies with larger sample sizes are required to confirm that pregnancy does not make a difference in the severity of COVID-19 vaccine side effects.

### 4.4. Side Effects in Breastfed Infants

Children can present side effects after the vaccination of their mother. We found that irritability was the most frequent self-reported symptom, followed by fever and diarrhea, in agreement with the literature [21,63]. CoronaVac registered the highest frequency of effects. McLaurin and colleagues did not show differences between mRNA-1273 and BNT162b2 [63], while Bertrand and colleagues found more drowsiness in children whose mothers received mRNA-1273 than BNT162b2 [21]. Vaccination protects the infant by transferring IgA and IgG antibodies and immune cells through the breast milk [19,22,58,64].

### 4.5. Limitations

Unfortunately, self-reported side effects could not be validated by a physician. The technique used for recruiting participants could have biased inclusion of contacts whom they knew had similar experiences. Furthermore, people that suffer from more symptoms might have been more interested in answering the survey. The recall bias was another limitation; participants could have remembered symptoms that have affected their health or their daily activities more, and some results could have been overestimated. The BNT162b2 and ChAdOx1 vaccines predominated in the study population, which was consistent with the greater availability of these vaccines in Mexico or in the neighboring country, the USA. It is necessary to continue with studies that include a greater number of participants who received Gam-COVID-Vac, Ad26.CoV2.S, and mRNA-1273. Results for breastfed infants were exploratory and conclusions are non-definitive due to the nonspecific nature of symptoms and small sample size. Further studies are needed to answer the unknowns that are left after this study.

## 5. Conclusions

Pregnant women had a tendency toward fewer systemic symptoms than non-pregnant/non-breastfeeding women after the first or only dose. BNT162b2 had a higher frequency of local symptoms in pregnancy. Lactating women experienced fewer local side effects after the first or single dose, but these were more frequent after the second dose. ChAdOx1 increased the chances of developing both local and systemic side effects after the first dose, but the opposite occurred after the second dose in lactating women. The severity was similar across groups, although the result of lack of association in pregnancy requires studies with a larger sample size. Irritability was the most reported symptom in breastfed infants. The present study contributes to the knowledge about the safety of COVID-19 vaccination during pregnancy and breastfeeding, which was one the main concerns against vaccination. This information can encourage women to obtain the vaccine, as these groups of the population are at higher risk for severe COVID-19.

## Figures and Tables

**Table 1:** Sociodemographic, medical, and vaccination history characteristics according to pregnancy and breastfeeding status. Mexican female population, August–September 2021 (*n* = 3167).

	Pregnancy and Breastfeeding Status			
	Non-Pregnant/ Non-Breastfeeding (*n* = 2694) *n* (%)	Pregnant (*n* = 110) *n* (%)	Breastfeeding (*n* = 363) *n* (%)	Chi-Square *p*-Value
Sociodemographic				
Age (years)				
<29	498 (18.5)	32 (29.1)	62 (17.1)	<0.001
30–39	1009 (37.5)	71 (64.5)	273 (75.2)	
40–49	649 (24.1)	7 (6.5)	28 (7.7)	
≥50	538 (20.0)	0 (0.0)	0 (0.0)	
Schooling				
Middle school	78 (2.9)	5 (4.5)	6 (1.7)	<0.001
High school	319 (11.8)	13 (11.8)	44 (11.7)	
Bachelor’s degree	1344 (49.9)	60 (54.5)	222 (61.2)	
Postgraduate	953 (35.4)	32 (29.1)	91 (25.1)	
Occupation				
Employed/self-employed	1876 (69.6)	81 (73.6)	248 (68.3)	<0.001
Housewife	352 (13.1)	18 (16.4)	100 (27.5)	
Retired/unemployed	214 (7.9)	4 (3.6)	7 (1.9)	
Student	252 (9.4)	7 (6.4)	8 (2.2)	
Risk factors and comorbidities				
Smoking	363 (13.5)	4 (3.6)	13 (3.6)	<0.001
Comorbidity (any)	566 (21.0)	10 (9.1)	42 (11.6)	<0.001
Overweight/obese	1379 (51.2)	63 (57.3)	164 (45.2)	0.037
Hypertension	198 (7.3)	2 (1.8)	7 (1.9)	<0.001
Prediabetes or diabetes	134 (5.0)	2 (1.8)	4 (1.1)	<0.001
Allergies	897 (33.3)	28 (25.5	113 (31.3)	0.178
Vaccination and vaccines				
COVID-19 before 1st dose	679 (25.2)	26 (23.6)	74 (20.4)	0.217
Preventive medication				
First dose	223 (8.3)	4 (3.6)	19 (5.2)	0.033
Second dose	111 (7.9)	3 (3.5)	4 (3.4)	0.078
Type of vaccine				
BNT162b2	1063 (39.5)	58 (52.7)	115 (31.7)	<0.001
ChAdOx1	787 (29.2)	20 (18.2)	133 (36.6)	
Ad5-nCoV	413 (15.3)	5 (4.5)	61 (16.8)	
CoronaVac	195 (7.2)	13 (11.8)	22 (6.1)	
Gam-COVID-Vac	129 (4.8)	14 (12.7)	22 (6.1)	
Ad26.CoV2.S	71 (2.6)	0 (0.0)	6 (1.7)	
mRNA-1273	36 (1.3)	0 (0.0)	4 (1.1)	

**Table 2:** Extension of COVID-19 vaccination side effects by type of vaccine in pregnant women.

First Dose (*n* = 105) ^b^						
Type of Vaccine ^b^	Absent	Local	Systemic	Local and Systemic	Chi-Square *p*-Value	Adjusted OR ^a^ (95% CI)
	*n* (%)	*n* (%)	*n* (%)	*n* (%)		
BNT162b2	19 (63.3)	14 (82.4)	1 (25.0)	24 (44.4)	0.006	1.0 (Ref)
ChAdOx1	1 (3.3)	2 (11.8)	2 (50.0)	15 (27.8)		5.1 (1.6, 16.8) *
CoronaVac	7 (23.3)	1 (5.9)	1 (25.0)	4 (7.4)		0.7 (0.2, 2.6)
Gam-COVID-Vac	3 (10.0)	0 (0.0)	0 (0.0)	11 (20.4)		4.9 (1.2, 20.4) *
Total	30 (100)	17 (100)	4 (100)	54 (100)		
Second Dose (*n* = 86)						
BNT162b2	19 (54.3)	9 (75.0)	2 (40.0)	24 (70.6)	0.068	1.0 (Ref)
ChAdOx1	6 (17.1)	0 (0.0)	0 (0.0)	2 (5.9)		0.25 (0.04, 1.8)
CoronaVac	6 (17.1)	2 (16.7)	3 (60.0)	2 (5.9)		0.9 (0.2, 3.0)
Gam-COVID-Vac	4 (11.4)	1 (8.3)	0 (0.0)	6 (17.6)		1.4 (0.3, 5.4)
Total	35 (100)	12 (100)	5 (100)	34 (100)		

* *p* < 0.01. ^a^ Adjusted for comorbidity, allergies, preventive use of medication to prevent symptoms before vaccination, and history of confirmed COVID-19 infection. ^b^ The frequency distribution of Ad5-nCoV (*n* = 5) was not included due to the small sample size.

**Table 3:** Extension of COVID-19 vaccination side effects by type of vaccine in breastfeeding women.

First or Single Dose (*n* = 353) ^b^						
Type of Vaccine	Absent	Local	Systemic	Local and Systemic	Chi-Square *p*-Value	Adjusted OR ^a^ (95% CI)
	*n* (%)	*n* (%)	*n* (%)	*n* (%)		
BNT162b2	31 (42.5)	19 (79.2)	0 (0.0)	65 (29.3)	<0.001	1.0 (Ref)
ChAdOx1	15 (20.5)	2 (8.3)	19 (55.9)	97 (43.7)		2.7 (1.6, 4.6) ***
Ad5-nCoV	15 (20.5)	1 (4.2)	9 (26.5)	36 (16.2)		1.4 (0.8, 2.6)
CoronaVac	10 (13.7)	0 (0.0)	2 (5.9)	10 (4.5)		0.6 (0.2 (1.5)
Gam-COVID-Vac	2 (2.7)	2 (8.3)	4 (11.8)	14 (6.3)		2.1 (0.8, 5.3)
Total	73 (100)	24 (100)	34 (100)	222 (100)		
Second Dose (*n* = 110) ^c^						
BNT162b2	17 (70.8)	19 (79.2)	9 (90.0)	45 (86.5)	0.323	1.0 (Ref)
ChAdOx1	3 (12.5)	4 (16.7)	0 (0.0)	1 (1.9)		0.2 (0.05, 0.7) *
Ad5-nCoV	2 (8.3)	0 (0.0)	0 (0.0)	2 (3.8)		0.5 (0.06, 4.4)
CoronaVac	2 (8.3)	1 (4.2)	1 (10.0)	4 (7.7)		0.9 (0.2, 3.8)
Total	24 (100)	24 (100)	10 (100)	52 (100)		

* *p* < 0.01, *** *p* < 0.001. ^a^ Adjusted for comorbidity, allergies, preventive use of medication to prevent symptoms before vaccination, and history of confirmed COVID-19 infection. ^b^ The frequency distributions of mRNA-1273 (*n* = 4) and Ad26.CoV2.S (*n* = 6) were not included due to the small sample sizes. ^c^ The frequency distributions of mRNA-1273 (*n* = 4), Ad26.CoV2.S (*n* = 1), and Gam-COVID-Vac (*n* = 1) were not included due to the small sample sizes.

**Table 4:** Severity of COVID-19 vaccination side effects after the first or only dose by pregnancy and breastfeeding status.

	Severity of Side Effect					
Status	Absent *n* (%)	Mild *n* (%)	Moderate/Severe *n* (%)	Total *n* (%)	Chi-Square *p*-Value	Adjusted OR ^a^ (95% CI)
Non-pregnant/non-breastfeeding	1185 (44.0)	794 (29.5)	715 (26.5)	2694 (100)	0.148	1.0 (Ref)
Pregnant	60 (54.5)	22 (20.0)	28 (25.5)	110 (100)		0.8 (0.5, 1.1)
Breastfeeding	155 (42.7)	114 (31.4)	94 (25.9)	363 (100)		1.1 (0.9, 1.3)

^a^ Adjusted for the preventive use of medication to prevent symptoms before vaccination and history of confirmed COVID-19 infection.

**Table 5:** Severity of COVID-19 vaccination side effects after the first or only dose by type of vaccine in pregnant and breastfeeding women.

Pregnant Women (*n* = 105) ^b^					
Type of Vaccine ^b^	Absent	Mild	Moderate/Severe	Chi-Square *p*-Value	Adjusted OR ^a^ (95% CI)
	*n* (%)	*n* (%)	*n* (%)		
BNT162b2	37 (63.8)	13 (59.1)	8 (32.0)	0.007	1.0 (Ref)
ChAdOx1	5 (8.6)	4 (18.2)	11 (44.0)		10.6 (3.39, 34) ***
CoronaVac	9 (15.5)	3 (13.6)	1 (4.0)		1.5 (0.35, 6.0)
Gam-COVID-Vac	7 (12.1)	2 (14.3)	5 (20.0)		3.47 (1.01, 11.9) *
Total	58 (100)	22 (100)	25 (100)		
Breastfeeding Women (*n* = 353) ^c^					
BNT162b2	70 (46.7)	33 (30.0)	12 (12.9)	<0.001	1.0 (Ref)
ChAdOx1	24 (16.0)	52 (47.3)	57 (61.3)		6.4 (3.9, 10.6) ***
Ad5-nCoV	33 (22.0)	13 (11.8)	15 (16.1)		1.7 (0.9, 3.2)
CoronaVac	15 (10.0)	4 (3.6)	3 (3.2)		0.8 (0.3, 2.1)
Gam-COVID-Vac	8 (5.3)	8 (7.3)	6 (6.5)		3.0 (1.3, 7.2) *
Total	150 (100)	110 (100)	93 (100)		

* *p* < 0.05, *** *p* < 0.001. ^a^ Adjusted for comorbidity, allergies, preventive use of medication to prevent symptoms before vaccination, and history of confirmed COVID-19 infection. ^b^ The frequency distribution of Ad5-nCoV (*n* = 5) was not included due to the small sample size, and was also excluded in pregnant women. ^c^ The frequency distributions of mRNA-1273 (*n* = 4) and Ad26.CoV2.S (*n* = 6) were not included due to the small sample sizes in pregnant and lactating women.

## Data Availability

The raw data supporting the conclusions of this article will be made available by the authors, without undue reservation.

## Supplementary Materials

The following supporting information can be downloaded at https://www.mdpi.com/article/10.3390/vaccines11081280/s1: Table S1. Variables description, Table S2. Extension of COVID-19 vaccination side effects according to number of doses and pregnancy status. Mexican female population, August - September 2021, Table S3. Extension of COVID-19 vaccination side effects according to number of doses and breastfeeding status. Mexican female population-August–September 2021, Table S4. Presentation of symptoms in breastfed infants by age and type of vaccine, and Table S5. Side effects reported by mothers in their breastfed infants after COVID-19 vaccination.

## References

[1] (2020). WHO COVID-19 Dashboard.

[2] Severe Acute Respiratory Syndrome Coronavirus 2 Isolate Wuhan-Hu-1, Co—Nucleotide—NCBI. https://www.ncbi.nlm.nih.gov/nuccore/MN908947.

[3] Camacho Moll M.E., Salinas Martinez A.M., Tovar Cisneros B., García Onofre J.I., Navarrete Floriano G., Bermúdez De León M. (2022). Extension and Severity of Self-Reported Side Effects of Seven COVID-19 Vaccines in Mexican Population. Front. Public Health.

[4] Rasmussen S.A., Jamieson D.J. (2022). COVID-19 and Pregnancy. Infect. Dis. Clin. N. Am..

[5] Golan Y., Prahl M., Cassidy A.G., Gay C., Wu A.H.B., Jigmeddagva U., Lin C.Y., Gonzalez V.J., Basilio E., Chidboy M.A. (2021). COVID-19 MRNA Vaccination in Lactation: Assessment of Adverse Events and Vaccine Related Antibodies in Mother-Infant Dyads. Front. Immunol..

[6] Low J.M., Gu Y., Ng M.S.F., Amin Z., Lee L.Y., Ng Y.P.M., Shunmuganathan B.D., Niu Y., Gupta R., Tambyah P.A. (2021). Codominant IgG and IgA Expression with Minimal Vaccine MRNA in Milk of BNT162b2 Vaccinees. NPJ Vaccines.

[7] Polack F.P., Thomas S.J., Kitchin N., Absalon J., Gurtman A., Lockhart S., Perez J.L., Pérez Marc G., Moreira E.D., Zerbini C. (2020). Safety and Efficacy of the BNT162b2 MRNA Covid-19 Vaccine. N. Engl. J. Med..

[8] Sadoff J., Gray G., Vandebosch A., Cárdenas V., Shukarev G., Grinsztejn B., Goepfert P.A., Truyers C., Fennema H., Spiessens B. (2021). Safety and Efficacy of Single-Dose Ad26.COV2.S Vaccine against COVID-19. N. Engl. J. Med..

[9] Ramasamy M.N., Minassian A.M., Ewer K.J., Flaxman A.L., Folegatti P.M., Owens D.R., Voysey M., Aley P.K., Angus B., Babbage G. (2020). Safety and Immunogenicity of ChAdOx1 NCoV-19 Vaccine Administered in a Prime-Boost Regimen in Young and Old Adults (COV002): A Single-Blind, Randomised, Controlled, Phase 2/3 Trial. Lancet.

[10] Zhu F.C., Guan X.H., Li Y.H., Huang J.Y., Jiang T., Hou L.H., Li J.X., Yang B.F., Wang L., Wang W.J. (2020). Immunogenicity and Safety of a Recombinant Adenovirus Type-5-Vectored COVID-19 Vaccine in Healthy Adults Aged 18 Years or Older: A Randomised, Double-Blind, Placebo-Controlled, Phase 2 Trial. Lancet.

[11] Halperin S.A., Ye L., MacKinnon-Cameron D., Smith B., Cahn P.E., Ruiz-Palacios G.M., Ikram A., Lanas F., Guerrero M.L., Navarro S.R.M. (2022). Final Efficacy Analysis, Interim Safety Analysis, and Immunogenicity of a Single Dose of Recombinant Novel Coronavirus Vaccine (Adenovirus Type 5 Vector) in Adults 18 Years and Older: An International, Multicentre, Randomised, Double-Blinded, Placebo-Controlled Phase 3 Trial. Lancet.

[12] Logunov D.Y., Dolzhikova I.V., Shcheblyakov D.V., Tukhvatulin A.I., Zubkova O.V., Dzharullaeva A.S., Kovyrshina A.V., Lubenets N.L., Grousova D.M., Erokhova A.S. (2021). Safety and Efficacy of an RAd26 and RAd5 Vector-Based Heterologous Prime-Boost COVID-19 Vaccine: An Interim Analysis of a Randomised Controlled Phase 3 Trial in Russia. Lancet.

[13] El Sahly H.M., Baden L.R., Essink B., Doblecki-Lewis S., Martin J.M., Anderson E.J., Campbell T.B., Clark J., Jackson L.A., Fichtenbaum C.J. (2021). Efficacy of the MRNA-1273 SARS-CoV-2 Vaccine at Completion of Blinded Phase. N. Engl. J. Med..

[14] Blakeway H., Prasad S., Kalafat E., Heath P.T., Ladhani S.N., Le Doare K., Magee L.A., O’Brien P., Rezvani A., von Dadelszen P. (2022). COVID-19 Vaccination during Pregnancy: Coverage and Safety. Am. J. Obstet. Gynecol..

[15] Wainstock T., Yoles I., Sergienko R., Sheiner E. (2021). Prenatal Maternal COVID-19 Vaccination and Pregnancy Outcomes. Vaccine.

[16] Gray K.J., Bordt E.A., Atyeo C., Deriso E., Akinwunmi B., Young N., Baez A.M., Shook L.L., Cvrk D., James K. (2021). Coronavirus Disease 2019 Vaccine Response in Pregnant and Lactating Women: A Cohort Study. Am. J. Obstet. Gynecol..

[17] Bookstein Peretz S., Regev N., Novick L., Nachshol M., Goffer E., Ben-David A., Asraf K., Doolman R., Gal Levin E., Regev Yochay G. (2021). Short-term Outcome of Pregnant Women Vaccinated with BNT162b2 MRNA COVID-19 Vaccine. Ultrasound Obstet. Gynecol..

[18] Shimabukuro T.T., Kim S.Y., Myers T.R., Moro P.L., Oduyebo T., Panagiotakopoulos L., Marquez P.L., Olson C.K., Liu R., Chang K.T. (2021). Preliminary Findings of MRNA Covid-19 Vaccine Safety in Pregnant Persons. N. Engl. J. Med..

[19] Collier A.R.Y., McMahan K., Yu J., Tostanoski L.H., Aguayo R., Ansel J., Chandrashekar A., Patel S., Apraku Bondzie E., Sellers D. (2021). Immunogenicity of COVID-19 MRNA Vaccines in Pregnant and Lactating Women. JAMA.

[20] Falsaperla R., Leone G., Familiari M., Ruggieri M. (2021). COVID-19 Vaccination in Pregnant and Lactating Women: A Systematic Review. Expert Rev. Vaccines.

[21] Bertrand K., Honerkamp-Smith G., Chambers C.D. (2021). Maternal and Child Outcomes Reported by Breastfeeding Women Following Messenger RNA COVID-19 Vaccination. Breastfeed. Med..

[22] Charepe N., Gonçalves J., Juliano A.M., Lopes D.G., Canhão H., Soares H., Serrano E.F. (2021). COVID-19 MRNA Vaccine and Antibody Response in Lactating Women: A Prospective Cohort Study. BMC Pregnancy Childbirth.

[23] Menni C., Klaser K., May A., Polidori L., Capdevila J., Louca P., Sudre C.H., Nguyen L.H., Drew D.A., Merino J. (2021). Vaccine Side-Effects and SARS-CoV-2 Infection after Vaccination in Users of the COVID Symptom Study App in the UK: A Prospective Observational Study. Lancet Infect. Dis..

[24] Menni C., Valdes A.M., Polidori L., Antonelli M., Penamakuri S., Nogal A., Louca P., May A., Figueiredo J.C., Hu C. (2022). Symptom Prevalence, Duration, and Risk of Hospital Admission in Individuals Infected with SARS-CoV-2 during Periods of Omicron and Delta Variant Dominance: A Prospective Observational Study from the ZOE COVID Study. Lancet.

[25] Antonelli M., Penfold R.S., Merino J., Sudre C.H., Molteni E., Berry S., Canas L.S., Graham M.S., Klaser K., Modat M. (2022). Risk Factors and Disease Profile of Post-Vaccination SARS-CoV-2 Infection in UK Users of the COVID Symptom Study App: A Prospective, Community-Based, Nested, Case-Control Study. Lancet. Infect. Dis..

[26] Rider L.G., Parks C.G., Wilkerson J., Schiffenbauer A.I., Kwok R.K., Noroozi Farhadi P., Nazir S., Ritter R., Sirotich E., Kennedy K. (2022). Baseline Factors Associated with Self-Reported Disease Flares Following COVID-19 Vaccination among Adults with Systemic Rheumatic Disease: Results from the COVID-19 Global Rheumatology Alliance Vaccine Survey. Rheumatology.

[27] Cheng Y., Li T., Zheng Y., Xu B., Bi Y., Hu Y., Zhou Y.H. (2022). Self-Reported Adverse Events among Chinese Healthcare Workers Immunized with COVID-19 Vaccines Composed of Inactivated SARS-CoV-2. Hum. Vaccin. Immunother..

[28] Li L., Robinson L.B., Patel R., Landman A.B., Fu X., Shenoy E.S., Hashimoto D.M., Banerji A., Wickner P.G., Samarakoon U. (2021). Association of Self-Reported High-Risk Allergy History With Allergy Symptoms After COVID-19 Vaccination. JAMA Netw. Open.

[29] Kadali R.A.K., Janagama R., Peruru S., Gajula V., Madathala R.R., Chennaiahgari N., Malayala S.V. (2021). Non-Life-Threatening Adverse Effects with COVID-19 MRNA-1273 Vaccine: A Randomized, Cross-Sectional Study on Healthcare Workers with Detailed Self-Reported Symptoms. J. Med. Virol..

[30] Yesuf E.A., Riad A., Sofi-Mahmudi A., Sudhakar M., Mekonnen A., Endalkachew S., Mama F., Muhidin S., Ayele B., Yahya M. (2022). Self-Reported Side Effects of the Oxford AstraZeneca COVID-19 Vaccine among Healthcare Workers in Ethiopia, Africa: A Cross-Sectional Study. Front. Public Health.

[31] Etemadifar M., Abhari A.P., Nouri H., Sigari A.A., Piran Daliyeh S.M., Maracy M.R., Salari M., Maleki S., Sedaghat N. (2022). Self-Reported Safety of the BBIBP-CorV (Sinopharm) COVID-19 Vaccine among Iranian People with Multiple Sclerosis. Hum. Vaccines Immunother..

[32] Kuodi P., Gorelik Y., Zayyad H., Wertheim O., Wiegler K.B., Abu Jabal K., Dror A.A., Nazzal S., Glikman D., Edelstein M. (2022). Association between BNT162b2 Vaccination and Reported Incidence of Post-COVID-19 Symptoms: Cross-Sectional Study 2020-21, Israel. NPJ Vaccines.

[33] Berg S.K., Wallach-Kildemoes H., Rasmussen L.R., Nygaard U., Bundgaard H., Petersen M.N.S., Hammer C.B., Ersbøll A.K., Thygesen L.C., Nielsen S.D. (2022). Short- and Long-Term Self-Reported Symptoms in Adolescents Aged 12–19 Years after Vaccination against SARS-CoV-2 Compared to Adolescents Not Vaccinated-A Danish Retrospective Cohort Study. Vaccines.

[34] Farland L.V., Khan S.M., Shilen A., Heslin K.M., Ishimwe P., Allen A.M., Herbst-Kralovetz M.M., Mahnert N.D., Pogreba-Brown K., Ernst K.C. (2022). COVID-19 Vaccination and Changes in the Menstrual Cycle among Vaccinated Persons. Fertil. Steril..

[35] Sultana A., Shahriar S., Tahsin M.R., Mim S.R., Fatema K.R., Saha A., Yesmin F., Bahar N.B., Samodder M., Mamun M.A.H. (2021). A Retrospective Cross-Sectional Study Assessing Self-Reported Adverse Events Following Immunization (AEFI) of the COVID-19 Vaccine in Bangladesh. Vaccines.

[36] Zare Z., Assarroudi A., Armat M.R., Laal Ahangar M., Estaji M., MoghaddamHosseini V., Dianatinasab M. (2022). Signs, Symptoms, and Side-Effects Presented by Different Types of COVID-19 Vaccines: A Prospective Cohort Study. Life.

[37] Deng L., Glover C., Dymock M., Pillsbury A., Marsh J.A., Quinn H.E., Leeb A., Cashman P., Snelling T.L., Wood N. (2022). The Short Term Safety of COVID-19 Vaccines in Australia: AusVaxSafety Active Surveillance, February–August 2021. Med. J. Aust..

[38] Hermann E.A., Lee B., Balte P.P., Xanthakis V., Kirkpatrick B.D., Cushman M., Oelsner E. (2022). Association of Symptoms After COVID-19 Vaccination With Anti-SARS-CoV-2 Antibody Response in the Framingham Heart Study. JAMA Netw. Open.

[39] Tissot N., Brunel A.-S., Bozon F., Rosolen B., Chirouze C., Bouiller K. (2021). Patients with History of Covid-19 Had More Side Effects after the First Dose of COVID-19 Vaccine. Vaccine.

[40] Serwaa D., Osei-Boakye F., Nkansah C., Ahiatrogah S., Lamptey E., Abdulai R., Antwi M.H., Wirekoh E.Y., Owusu E., Buckman T.A. (2021). Non-Life-Threatening Adverse Reactions from COVID-19 Vaccine; a Cross-Sectional Study with Self-Reported Symptoms among Ghanaian Healthcare Workers. Hum. Vaccines Immunother..

[41] Kadali R.A.K., Janagama R., Peruru S.R., Racherla S., Tirumala R., Madathala R.R., Gajula V. (2021). Adverse Effects of COVID-19 Messenger RNA Vaccines among Pregnant Women: A Cross-Sectional Study on Healthcare Workers with Detailed Self-Reported Symptoms. Am. J. Obstet. Gynecol..

[42] Sánchez-Saez F., Peiró S., Cuenca L., Vanaclocha H., Limón R., Salas D., Burgos J.S., Sánchez-Payá J., Meneu R., Díez J. (2022). Side Effects during the Week after First Dose Vaccination with Four COVID-19 Vaccines. Results of the ProVaVac Survey Study with 13,837 People in Spain. Vaccine.

[43] Perl S.H., Uzan-Yulzari A., Klainer H., Asiskovich L., Youngster M., Rinott E., Youngster I. (2021). SARS-CoV-2-Specific Antibodies in Breast Milk after COVID-19 Vaccination of Breastfeeding Women. JAMA J. Am. Med. Assoc..

[44] Muyldermans J., De Weerdt L., De Brabandere L., Maertens K., Tommelein E. (2022). The Effects of COVID-19 Vaccination on Lactating Women: A Systematic Review of the Literature. Front. Immunol..

[45] Low J.M., Lee L.Y., Ng Y.P.M., Zhong Y., Amin Z. (2022). Breastfeeding Mother and Child Clinical Outcomes after COVID-19 Vaccination. J. Hum. Lact..

[46] Kachikis A., Englund J.A., Singleton M., Covelli I., Drake A.L., Eckert L.O. (2021). Short-Term Reactions Among Pregnant and Lactating Individuals in the First Wave of the COVID-19 Vaccine Rollout. JAMA Netw. Open.

[47] Pietrasanta C., Ronchi A., Crippa B.L., Artieri G., Ballerini C., Crimi R., Mosca F., Pugni L. (2022). Coronavirus Disease 2019 Vaccination During Pregnancy and Breastfeeding: A Review of Evidence and Current Recommendations in Europe, North America, and Australasia. Front. Pediatr..

[48] Takahashi K., Samura O., Hasegawa A., Okubo H., Morimoto K., Horiya M., Okamoto A., Ochiai D., Tanaka M., Sekiguchi M. (2023). COVID-19 MRNA Vaccination Status and Concerns among Pregnant Women in Japan: A Multicenter Questionnaire Survey. BMC Pregnancy Childbirth.

[49] Özen D.S.K., Kiraz A.K., Yurt Ö.F., Kiliç I.Z., Demirağ M.D. (2022). COVID-19 Vaccination Rates and Factors Affecting Vaccine Hesitancy among Pregnant Women during the Pandemic Period in Turkey: A Single-Center Experience. Vaccines.

[50] Colciago E., Capitoli G., Vergani P., Ornaghi S. (2022). Women’s Attitude towards COVID-19 Vaccination in Pregnancy: A Survey Study in Northern Italy. Int. J. Gynaecol. Obstet..

[51] Egloff C., Couffignal C., Cordier A.G., Deruelle P., Sibiude J., Anselem O., Benachi A., Luton D., Mandelbrot L., Vauloup-Fellous C. (2022). Pregnant Women’s Perceptions of the COVID-19 Vaccine: A French Survey. PLoS ONE.

[52] Skirrow H., Barnett S., Bell S., Riaposova L., Mounier-Jack S., Kampmann B., Holder B. (2022). Women’s Views on Accepting COVID-19 Vaccination during and after Pregnancy, and for Their Babies: A Multi-Methods Study in the UK. BMC Pregnancy Childbirth.

[53] Bae S., Lee Y.W., Lim S.Y., Lee J.H., Lim J.S., Lee S., Park S., Kim S.K., Lim Y.J., Kim E.O. (2021). Adverse Reactions Following the First Dose of ChAdOx1 NCoV-19 Vaccine and BNT162b2 Vaccine for Healthcare Workers in South Korea. J. Korean Med. Sci..

[54] Lee Y.W., Lim S.Y., Lee J.H., Lim J.S., Kim M., Kwon S., Joo J., Kwak S.H., Kim E.O., Jung J. (2021). Adverse Reactions of the Second Dose of the BNT162b2 MRNA COVID-19 Vaccine in Healthcare Workers in Korea. J. Korean Med. Sci..

[55] Harris C.V., Bradlyn A.S., Coffman J., Gunel E., Cottrell L. (2008). BMI-Based Body Size Guides for Women and Men: Development and Validation of a Novel Pictorial Method to Assess Weight-Related Concepts. Int. J. Obes..

[56] Allotey J., Stallings E., Bonet M., Yap M., Chatterjee S., Kew T., Debenham L., Llavall A.C., Dixit A., Zhou D. (2020). Clinical Manifestations, Risk Factors, and Maternal and Perinatal Outcomes of Coronavirus Disease 2019 in Pregnancy: Living Systematic Review and Meta-Analysis. BMJ.

[57] Lokken E.M., Huebner E.M., Taylor G.G., Hendrickson S., Vanderhoeven J., Kachikis A., Coler B., Walker C.L., Sheng J.S., al-Haddad B.J.S. (2021). Disease Severity, Pregnancy Outcomes, and Maternal Deaths among Pregnant Patients with Severe Acute Respiratory Syndrome Coronavirus 2 Infection in Washington State. Am. J. Obstet. Gynecol..

[58] Laguila Altoé A., Marques Mambriz A.P., Cardozo D.M., Valentini Zacarias J.M., Laguila Visentainer J.E., Bahls-Pinto L.D. (2022). Vaccine Protection Through Placenta and Breastfeeding: The Unmet Topic in COVID-19 Pandemic. Front. Immunol..

[59] Wilson S.L., Wiysonge C. (2020). Social Media and Vaccine Hesitancy. BMJ Glob. Health.

[60] Skjefte M., Ngirbabul M., Akeju O., Escudero D., Hernandez-Diaz S., Wyszynski D.F., Wu J.W. (2021). COVID-19 Vaccine Acceptance among Pregnant Women and Mothers of Young Children: Results of a Survey in 16 Countries. Eur. J. Epidemiol..

[61] Hagrass A.I., Almadhoon H.W., Al-kafarna M., Almaghary B.K., Nourelden A.Z., Fathallah A.H., Hasan M.T., Mohammed Y.A., Al-Nabahin A.O., Wafi D.S. (2022). Maternal and Neonatal Safety Outcomes after SAR-CoV-2 Vaccination during Pregnancy: A Systematic Review and Meta-Analysis. BMC Pregnancy Childbirth.

[62] Goldshtein I., Nevo D., Steinberg D.M., Rotem R.S., Gorfine M., Chodick G., Segal Y. (2021). Association Between BNT162b2 Vaccination and Incidence of SARS-CoV-2 Infection in Pregnant Women. JAMA.

[63] McLaurin-Jiang S., Garner C.D., Krutsch K., Hale T.W. (2021). Maternal and Child Symptoms Following COVID-19 Vaccination Among Breastfeeding Mothers. Breastfeed. Med..

[64] Blumberg D., Sridhar A., Lakshminrusimha S., Higgins R.D., Saade G. (2021). COVID-19 Vaccine Considerations during Pregnancy and Lactation. Am. J. Perinatol..

