# Supplementary material for: Side Effects of COVID-19 Vaccines in Pregnant and Lactating Mexican Women and Breastfed Infants: A Survey-Based Study
Source: Vaccines (Basel). 2023 Jul 25;11(8):1280. doi: 10.3390/vaccines11081280 (PMC10459654; doi:10.3390/vaccines11081280)
Supplement: Supplementary file 1 [file vaccines-11-01280-s001.zip › vaccines-2428134-supplementary.pdf]

Table S1. Variables description.

Variable	Recorded answers
Vaccine type	BNT162b2, ChAdOx1, Ad5-nCoV, CoronaVac, Gam-COVID-Vac, Ad26.CoV2.S, or ARNm-1273
Vaccine doses	1 or 2
Medication prior to vaccination	Yes or no
Presentation of side effects	Yes or no
If yes, type of side effects	Local: Arm pain, Injection site pain, Injection site swelling, Injection site itching, Injection site redness<br>Systemic: Headache, Muscle pain, Fatigue or tiredness, Lack of energy, Fever, Desire to sleep, Chills, Malaise, Bone or joint pain, Nausea, Dizziness, Hot flashes, Eye movement pain, Sweating, Sore throat, Stuffy nose, Chest pain, Diarrhea, A faster or lower heartbeat, Irritated eyes, Running nose, Difficulty breathing or dyspnea, Abdominal pain, Lymph nodes tenderness or swelling, Cough, Rise in blood pressure, Vomiting, Lower blood pressure, Skin hives, Face and neck swelling, Bleeding, Skin spots, Loss of consciousness, Numbness, Mental symptoms, Spasms, General itchiness, Platelet effects, General swelling
Extension of side effects categorization	None, Local, Systemic, Both
Took medicine to relieve symptoms	Yes or no
Suspended daily activities or missed work	Yes or no
Sought medical attention (outpatient or emergency care)	Yes or no
Hospitalization	Yes or no
Severity of side effects categorization	Absent (did not experience side effects)<br>Mild: Took medicine to relieve symptoms, but did not suspend daily activities, miss work, seek medical attention, or was hospitalized<br>Moderate: Took medicine to relieve symptoms, did suspend daily activities or missed work, but did not seek medical attention, or was hospitalized<br>Severe: Did seek medical attention or was hospitalized, regardless of the need to take medication to relieve symptoms, suspension of activities or missed work
Comorbidities	Yes or no
If yes, type of comorbidity	Diabetes, High blood pressure, Renal disease, Chronic obstructive pulmonary disease, Asthma, Immunological disease, Cerebrovascular disease, cardiovascular disease, Hepatic disease.
Schooling	Middle school, High school, Bachelor's degree, or Postgraduate
History of allergies	Yes or no
History of COVID-19 disease	Yes or no
Occupation	Employed, Self-employed, Housewife, Retired, Unemployed, or Student
Tobacco smoking	Yes or no
Nutritional status	Low weight, Normal weight, Overweight, or Obese
Age	Adults (years) / Infants (months)
Sex	Male or Female

Table S2. Extension of COVID-19 vaccination side effects according to number of doses and pregnancy status. Mexican female population, August - September 2021.

	First Dose (n=2,804)					Second dose (n=1,492)				
	Pregnant (n = 110)		Non-pregnant/non-breastfeeding (n = 2,694)			Pregnant (n =86)		Non-pregnant/non-breastfeeding (n = 1,405)		
	**n**	**% (95% CI)**	**n**	**% (95% CI)**	**Chi-square or Fisher's *p* value**	**n**	**% (95% CI)**	**n**	**% (95% CI)**	**Chi-square or Fisher's *p* value**
Extension										
No symptoms	31	28.18 (20.6-37.2)	602	22.3 (20.8, 24)		35	40.7 (30.9-51.3)	522	37.2 (34.7, 39.7)	
Local symptoms	17	15.45 (9.9-23.4)	301	11.2 (10, 12.4)	0.063	12	13.95 (8.2-22.8)	176	12.5 (10.9, 14.4)	0.557
Systemic symptoms	4	3.64 (1.4-9)	248	9.2 (8.2, 10.4)		5	5.81 (2.5-12.9)	146	10.4 (8.9, 12.1)	
Both, local and systemic symptoms	58	52.73 (43.5-61.8)	1,543	57.3 (55.4, 59.1)		34	39.53 (29.9-50.1)	561	39.9 (37.4, 42.5)	
Type of local side effect										
Arm pain	63	57.27 (47.9-66.1)	1,455	54 (52.1, 55.9)	0.495	38	44.19 (34.2-54.7)	562	40 (37.5, 42.6)	0.439
Injection site pain	49	44.55 (35.6-53.9)	1,268	47.1 (45.2, 49)	0.608	27	31.4 (22.6-41.8)	434	30.9 (28.5, 33.4)	0.918
Injection site swelling	6	5.45 (2.5-11.4)	255	9.5 (8.4, 10.6)	0.156	2	2.33 (0.6-8.1)	79	5.6 (4.5, 7)	0.321[a]321[a]
Injection site itching	6	5.45 (2.5-11.4)	138	5.1 (4.4, 6)	0.876	1	1.16 (0.2-6.3)	35	2.5 (1.8, 3.4)	0.719[a]719[a]
Injection site redness	3	2.73 (0.9-7.7)	126	4.7 (3.9, 5.5)	0.339	0	0 (0-4.3)	38	2.7 (2, 3.7)	0.165[a]165[a]
Type of systemic side effect										
Headache	34	30.91 (23-40.1)	1,083	40.17 (38.3, 42)	0.052	23	26.74 (18.5-36.9)	385	27.4 (25.1, 29.8)	0.897
Muscle pain	26	23.64 (16.7-32.4)	910	33.75 (32, 35.6)	0.027	13	15.12 (9.1-24.2)	273	19.4 (17.4, 21.6)	0.325
Fatigue or tiredness	20	18.18 (12.1-26.4)	768	28.5 (26.8, 30.2)	0.018	21	24.42 (16.6-34.5)	334	23.8 (21.6, 26.1)	0.888
Lack of energy	24	21.82 (15.1-30.4)	774	28.7 (27.1, 30.5)	0.116	14	16.28 (10-25.5)	214	15.2 (13.4, 17.2)	0.791
Fever	10	9.09 (5-15.9)	652	24.2 (22.6, 25.9)	0.000	10	11.63 (6.4-20.1)	163	11.6 (10, 13.4)	0.992

	First Dose (n=2,804)					Second dose (n=1,492)				
	Pregnant (n = 110)		Non-pregnant/non-breastfeeding (n = 2,694)			Pregnant (n =86)		Non-pregnant/non-breastfeeding (n = 1,405)		
	n	% (95% CI)	n	% (95% CI)	Chi-square or Fisher's *p* value	n	% (95% CI)	n	% (95% CI)	Chi-square or Fisher's *p* value
Desire to sleep	26	23.64 (16.7-32.4)	653	24.2 (22.7, 25.9)	0.888	14	16.28 (10-25.5)	212	15.1 (13.3, 17.1)	0.763
Chills	16	14.55 (9.2-22.3)	586	21.8 (20.2, 23.3)	0.072	8	9.3 (4.8-17.3)	158	11.2 (9.7, 13)	0.580
Malaise	12	10.91 (6.4-18.1)	540	20 (18.6, 21.6)	0.018	10	11.63 (6.4-20.1)	188	13.4 (11.7, 15.3)	0.644
Bone or joint pain	14	12.73 (7.7-20.2)	514	19.1 (17.6, 20.6)	0.095	6	6.98 (3.2-14.4)	163	11.6 (10, 13.4)	0.190
Nausea	5	4.55 (2-10.2)	203	7.5 (6.6, 8.6)	0.242	6	6.98 (3.2-14.4)	49	3.5 (2.6, 4.6)	0.128[a]
Dizziness	2	1.82 (0.5-6.4)	191	7.1 (6.2, 8.1)	0.032	2	2.33 (0.6-8.1)	59	4.2 (3.3, 5.4)	0.576[a]
Hot flashes	2	1.82 (0.5-6.4)	185	6.9 (6, 7.9)	0.038	1	1.16 (0.2-6.3)	45	3.2 (2.4, 4.3)	0.515[a]
Eye movement pain	5	4.55 (2-10.2)	177	6.6 (5.7, 7.6)	0.399	4	4.65 (1.8-11.4)	37	2.6 (1.9, 3.6)	0.292[a]
Sweating	3	2.73 (0.9-7.7)	173	6.4 (5.6, 7.4)	0.118	3	3.49 (1.2-9.8)	39	2.8 (2, 3.8)	0.731[a]
Sore throat	2	1.82 (0.5-6.4)	146	5.4 (4.6, 6.3)	0.098	2	2.33 (0.6-8.1)	51	3.6 (2.8, 4.7)	0.765[a]
Stuffy nose	2	1.82 (0.5-6.4)	147	5.5 (4.7, 6.4)	0.096	6	6.98 (3.2-14.4)	42	3 (2.2, 4)	0.054[a]
Chest pain	0	0 (0-3.4)	137	5.1 (4.3, 6)	0.015	0	0 (0-4.3)	30	2.1 (1.5, 3)	0.413[a]
Diarrhea	4	3.64 (1.4-9)	128	4.8 (4, 5.6)	0.589	3	3.49 (1.2-9.8)	52	3.7 (2.8, 4.8)	1.000[a]
A faster or lower heartbeat	3	2.73 (0.9-7.7)	135	5 (4.2, 5.9)	0.278	0	0 (0-4.3)	31	2.2 (1.6, 3.1)	0.254[a]
Irritated eyes	1	0.91 (0.2-5)	89	3.3 (2.7, 4)	0.263[a]	0	0 (0-4.3)	23	1.6 (1.1, 2.4)	0.639[a]
Running nose	2	1.82 (0.5-6.4)	88	3.3 (2.7, 4)	0.582[a]	3	3.49 (1.2-9.8)	39	2.8 (2, 3.8)	0.731[a]
Difficulty breathing (dyspnea)	1	0.91 (0.2-5)	82	3 (2.5, 3.8)	0.379[a]	0	0 (0-4.3)	16	1.1 (0.7, 1.8)	0.445[a]
Abdominal pain	1	0.91 (0.2-5)	73	2.7 (2.2, 3.4)	0.367[a]	0	0 (0-4.3)	23	1.6 (1.1, 2.4)	0.639[a]
Lymph nodes tenderness or swelling	1	0.91 (0.2-5)	67	2.5 (2, 3.1)	0.521[a]	0	0 (0-4.3)	32	2.3 (1.6, 3.2)	0.255[a]
Cough	1	0.91 (0.2-5)	70	2.6 (2.1, 3.3)	0.527[a]	2	2.33 (0.6-8.1)	24	1.7 (1.2, 2.5)	0.659[a]

	First Dose (n=2,804)					Second dose (n=1,492)				
	Pregnant (n = 110)		Non-pregnant/non-breastfeeding (n = 2,694)			Pregnant (n =86)		Non-pregnant/non-breastfeeding (n = 1,405)		
	**n**	**% (95% CI)**	**n**	**% (95% CI)**	**Chi-square or Fisher's *p* value**	**n**	**% (95% CI)**	**n**	**% (95% CI)**	**Chi-square or Fisher's *p* value**
Rise in blood pressure	1	0.91 (0.2-5)	60	2.2 (1.7, 2.9)	0.732[a]	0	0 (0-4.3)	10	0.7 (0.4, 1.3)	1.000[a]
Vomiting	2	1.82 (0.5-6.4)	45	1.7 (1.3, 2.2)	0.707[a]	1	1.16 (0.2- 6.3)	14	1 (0.6, 1.7)	0.592[a]
Lower blood pressure	0	0 (0-3.4)	38	1.4 (1, 1.9)	0.402[a]	0	0 (0-4.3)	6	0.4 (0.2, 0.9)	1.000[a]
Skin hives	1	0.91 (0.2-5)	30	1.1 (0.8, 1.6)	1.000[a]	0	0 (0-4.3)	5	0.4 (0.2, 0.8)	1.000[a]
Face and neck swelling	1	0.91 (0.2-5)	20	0.7 (0.5, 1.1)	1.000[a]	0	0 (0-4.3)	4	0.3 (0.1, 0.7)	1.000[a]
Bleeding	0	0 (0-3.4)	17	0.6 (0.4, 1)	1.000[a]	0	0 (0-4.3)	1	0.1 (0, 0.4)	1.000[a]
Skin spots	0	0 (0-3.4)	14	0.5 (0.3, 0.9)	1.000[a]	0	0 (0-4.3)	2	0.1 (0, 0.5)	1.000[a]
Loss of consciousness	0	0 (0-3.4)	12	0.45 (0.3, 0.8)	1.000[a]	0	0 (0-4.3)	3	0.2 (0.1, 0.6)	1.000[a]
Numbness	0	0 (0-3.4)	12	0.4 (0.3, 0.8)	1.000[a]	0	0 (0-4.3)	4	0.3 (0.1, 0.7)	1.000[a]
Mental symptoms	0	0 (0-3.4)	6	0.2 (0.1, 0.5)	1.000[a]	0	0 (0-4.3)	2	0.1 (0, 0.5)	1.000[a]

[a] This data was analyzed by Fisher's exact test.

Table S3. Extension of COVID-19 vaccination side effects according to number of doses and breastfeeding status. Mexican female population-August - September 2021.

	First or only dose (3,057)					Second dose (1,522)				
	Breastfeeding (n=363)		Non-pregnant/non-breastfeeding (n= 2,694)		Chi-square or Fisher's *p* value	Breastfeeding (n=116)		Non-pregnant/non-breastfeeding (n=1,406)		Chi-square or Fisher's *p* value
	n	% (95% CI)	n	% (95% CI)		n	% (95% CI)	n	% (95% CI)	
Extension										
No symptoms	76	20.94 (17.1-25.4)	6022	22.3 (20.8, 24)	0.040	24	20.69 (14.3-28.9)	5222	37.2 (34.7, 39.7)	<0.0011
Local symptoms	24	6.61 (4.5-9.6)	301	11.2 (10, 12.4)		25	21.55 (15-29.9)	176	12.5 (10.9, 14.4)	
Systemic symptoms	35	9.64 (7-13.1)	248	9.2 (8.2, 10.4)		10	8.62 (4.7-15.1)	146	10.4 (8.9, 12.1)	
Local and systemic symptoms	228	62.81 (57.7-67.6)	1,543	57.3 (55.4, 59.1)		57	49.14 (40.2-58.1)	561	39.9 (37.4, 42.5)	
Type of local side effect										
Arm pain	202	55.65 (50.5-60.7)	1,455	54 (52.1, 55.9)	0.547	68	58.62 (49.5-67.2)	562	40 (37.5, 42.6)	<0.0011
Injection site pain	172	47.38 (42.3-52.5)	1,268	47.1 (45.2, 49)	0.900	49	42.24 (33.6-51.3)	434	30.9 (28.5, 33.4)	0.011
Injection site swelling	32	8.82 (6.3-12.2)	255	9.5 (8.4, 10.6)	0.693	10	8.62 (4.7-15.1)	79	5.6 (4.5, 7)	0.185
Injection site itching	23	6.34 (4.3-9.3)	138	5.1 (4.4, 6)	0.329	5	4.31 (1.9-9.7)	35	2.5 (1.8, 3.4)	0.239
Injection site redness	18	4.96 (3.2-7.7)	126	4.7 (3.9, 5.5)	0.810	5	4.31 (1.9-9.7)	38	2.7 (2, 3.7)	0.315
Type of systemic side effect										
Headache	169	46.56 (41.5-51.7)	1,083	40.17 (38.3, 42)	0.020	41	35.34 (27.2-44.4)	385	27.4 (25.1, 29.8)	0.066
Muscle pain	141	38.84 (34-43.9)	910	33.75 (32, 35.6)	0.055	27	23.28 (16.5-31.7)	273	19.4 (17.4, 21.6)	0.315
Fatigue or tiredness	125	34.44 (29.7-39.5)	768	28.5 (26.8, 30.2)	0.019	37	31.9 (24.1-40.8)	334	23.8 (21.6, 26.1)	0.050
Lack of energy	116	31.96 (27.4-36.9)	774	28.7 (27.1, 30.5)	0.201	22	18.97 (12.9-27)	214	15.2 (13.4, 17.2)	0.284
Fever	90	24.79 (20.6-29.5)	652	24.2 (22.6, 25.9)	0.799	11	9.48 (5.4-16.2)	163	11.6 (10, 13.4)	0.492
Desire to sleep	78	21.49 (17.6-26)	653	24.2 (22.7, 25.9)	0.252	22	18.97 (12.9-27)	212	15.1 (13.3, 17.1)	0.265
Chills	86	23.69 (19.6-28.3)	586	21.8 (20.2, 23.3)	0.398	7	6.03 (3-11.9)	158	11.2 (9.7, 13)	0.083

	First or only dose (3,057)					Second dose (1,522)				
	Breastfeeding (n=363)		Non-pregnant/non-breastfeeding (n= 2,694)		Chi-square or Fisher's *p* value	Breastfeeding (n=116)		Non-pregnant/non-breastfeeding (n=1,406)		Chi-square or Fisher's *p* value
	n	% (95% CI)	n	% (95% CI)		n	% (95% CI)	n	% (95% CI)	
Malaise	80	22.04 (18.1-26.6)	540	20 (18.6, 21.6)	0.371	16	13.79 (8.7-21.2)	188	13.4 (11.7, 15.3)	0.898
Bone or joint pain	86	23.69 (19.6-28.3)	514	19.1 (17.6, 20.6)	0.037	16	13.79 (8.7-21.2)	163	11.6 (10, 13.4)	0.480
Nausea	43	11.85 (8.9-15.6)	203	7.5 (6.6, 8.6)	0.005	6	5.17 (2.4-10.8)	49	3.5 (2.6, 4.6)	0.050[a]
Dizziness	42	11.57 (8.7-15.3)	191	7.1 (6.2, 8.1)	0.002	9	7.76 (4.1-14.1)	59	4.2 (3.3, 5.4)	0.074
Hot flashes	29	7.99 (5.6-11.2)	185	6.9 (6, 7.9)	0.429	4	3.45 (1.3-8.5)	45	3.2 (2.4, 4.3)	0.785[a]
Eye movement pain	33	9.09 (6.5-12.5)	177	6.6 (5.7, 7.6)	0.074	4	3.45 (1.3-8.5)	37	2.6 (1.9, 3.6)	0.548[a]
Sweating	26	7.16 (4.9-10.3)	173	6.4 (5.6, 7.4)	0.589	4	3.45 (1.3-8.5)	39	2.8 (2, 3.8)	0.564[a]
Sore throat	21	5.79 (3.8-8.7)	146	5.4 (4.6, 6.3)	0.771	6	5.17 (2.4-10.8)	51	3.6 (2.8, 4.7)	0.439[a]
Stuffy nose	17	4.68 (2.9-7.4)	147	5.5 (4.7, 6.4)	0.541	5	4.31 (1.9-9.7)	42	3 (2.2, 4)	0.399[a]
Chest pain	20	5.51 (3.6-8.4)	137	5.1 (4.3, 6)	0.729	2	1.72 (0.5-6.1)	30	2.1 (1.5, 3)	1.000[a]
Diarrhea	14	3.86 (2.3-6.4)	128	4.8 (4, 5.6)	0.449	6	5.17 (2.4-10.8)	52	3.7 (2.8, 4.8)	0.444[a]
A faster or lower heartbeat	11	3.03 (1.7-5.3)	135	5 (4.2, 5.9)	0.097	4	3.45 (1.3-8.5)	31	2.2 (1.6, 3.1)	0.335[a]
Irritated eyes	16	4.41 (2.7-7)	89	3.3 (2.7, 4)	0.277	0	0 (0-3.2)	23	1.6 (1.1, 2.4)	0.251[a]
Running nose	12	3.31 (1.9-5.7)	88	3.3 (2.7, 4)	0.967	1	0.86 (0.2-4.7)	39	2.8 (2, 3.8)	0.361[a]
Difficulty breathing (dyspnea)	10	2.75 (1.5-5)	82	3 (2.5, 3.8)	0.764	2	1.72 (0.5-6.1)	16	1.1 (0.7, 1.8)	0.642[a]
Abdominal pain	11	3.03 (1.7-5.3)	73	2.7 (2.2, 3.4)	0.724	0	0 (0-3.2)	23	1.6 (1.1, 2.4)	0.251[a]
Lymph nodes tenderness or swelling	13	3.58 (2.1-6)	67	2.5 (2, 3.1)	0.219	2	1.72 (0.5-6.1)	32	2.3 (1.6, 3.2)	1.000[a]
Cough	10	2.75 (1.5-5)	70	2.6 (2.1, 3.3)	0.859	0	0 (0-3.2)	24	1.7 (1.2, 2.5)	0.251[a]
Rise in blood pressure	9	2.48 (1.3-4.6)	60	2.2 (1.7, 2.9)	0.760	0	0 (0-3.2)	10	0.7 (0.4, 1.3)	1.000[a]
Vomiting	7	1.93 (0.9-3.9)	45	1.7 (1.3, 2.2)	0.720	3	2.59 (0.9-7.3)	14	1 (0.6, 1.7)	0.134[a]
Lower blood pressure	3	0.83 (0.3-2.4)	38	1.4 (1, 1.9)	0.472[a]	0	0 (0-3.2)	6	0.4 (0.2, 0.9)	1.000[a]

	First or only dose (3,057)					Second dose (1,522)				
	Breastfeeding (n=363)		Non-pregnant/non-breastfeeding (n= 2,694)		Chi-square or Fisher's *p* value	Breastfeeding (n=116)		Non-pregnant/non-breastfeeding (n=1,406)		Chi-square or Fisher's *p* value
	n	% (95% CI)	n	% (95% CI)		n	% (95% CI)	n	% (95% CI)	
Skin hives	0	0 (0-1)	30	1.1 (0.8, 1.6)	0.042[a]	1	0.86 (0.2-4.7)	5	0.4 (0.2, 0.8)	1.000[a]
Face and neck swelling	3	0.83 (0.3-2.4)	20	0.7 (0.5, 1.1)	0.749[a]	1	0.86 (0.2-4.7)	4	0.3 (0.1, 0.7)	0.328[a]
Bleeding	1	0.28 (0-1.5)	17	0.6 (0.4, 1)	0.714[a]	0	0 (0-3.2)	1	0.1 (0, 0.4)	1.000[a]
Skin spots	1	0.28 (0-1.5)	14	0.5 (0.3, 0.9)	1.000[a]	1	0.86 (0.2-4.7)	2	0.1 (0, 0.5)	0.212[a]
Loss of consciousness	2	0.55 (0.2-2)	12	0.45 (0.3, 0.8)	0.678[a]	2	1.72 (0.5-6.1)	3	0.2 (0.1, 0.6)	0.050[a]
Numbness	0	0 (0-1)	12	0.4 (0.3, 0.8)	0.381[a]	0	0 (0-3.2)	4	0.3 (0.1, 0.7)	1.000[a]
Mental symptoms	0	0 (0-1)	6	0.2 (0.1, 0.5)	1.000[a]	0	0 (0-3.2)	2	0.1 (0, 0.5)	1.000[a]
Spasms	0	0 (0-1)	2	0.1 (0, 0.3)	1.000[a]	0	0 (0-3.2)	2	0.1 (0, 0.5)	1.000[a]

[a] This data was analyzed by Fisher's exact test

Table S4. Presentation of symptoms in breastfed infants by age and type of vaccine.

	Symptom presentation		
	Total (n=363)	No (n=323)	Yes (n=40)
		n (%)	n (%)
Age of infant			
0-2 months	29	26 (89.7)	3 (10.3)
2.1-4 months	25	21 (84)	4 (16)
4.1-6 months	25	18 (72)	7 (28)
6.1 and older	284	258 (90.8)	26 (9.2)
Type of vaccine			
BNT162b2	115	107 (93)	8 (7)
ChAdOx1	133	111 (83.5)	22 (16.5)
Ad5-nCoV	61	58 (95.1)	3 (4.9)
CoronaVac	22	17 (77.3)	5 (22.7)
Gam-COVID-Vac	22	21 (95.5)	1 (4.5)
ARNm-1273	4	3 (75)	1 (25)
Ad26.CoV2.S	6	6 (1.9)	0 (0.0)

Table S5. Side effects reported by mothers in their breastfed infants after COVID-19 vaccination.

Symptoms	Total (n=363)
Irritability	12
Fever	9
Diarrhoea	9
Desire to sleep	6
Malaise	5
Vomit	4
Running/stuffy nose	3
Lack of energy	2
Cough	2
General body pain	1
Flu like symptoms	1
Breathing difficulty	1
Nasal bleeding	1
